# Contribution to pathogenesis of accessory proteins of deadly human coronaviruses

**DOI:** 10.3389/fcimb.2023.1166839

**Published:** 2023-05-01

**Authors:** Jesus Hurtado-Tamayo, Ricardo Requena-Platek, Luis Enjuanes, Melissa Bello-Perez, Isabel Sola

**Affiliations:** Department of Molecular and Cell Biology, National Center of Biotechnology (CNB-CSIC), Campus Universidad Autónoma de Madrid, Madrid, Spain

**Keywords:** coronavirus, SARS-CoV, MERS-CoV, SARS-CoV-2, accessory proteins, pathogenesis, innate immune response

## Abstract

Coronaviruses (CoVs) are enveloped and positive-stranded RNA viruses with a large genome (∼ 30kb). CoVs include essential genes, such as the replicase and four genes coding for structural proteins (S, M, N and E), and genes encoding accessory proteins, which are variable in number, sequence and function among different CoVs. Accessory proteins are non-essential for virus replication, but are frequently involved in virus-host interactions associated with virulence. The scientific literature on CoV accessory proteins includes information analyzing the effect of deleting or mutating accessory genes in the context of viral infection, which requires the engineering of CoV genomes using reverse genetics systems. However, a considerable number of publications analyze gene function by overexpressing the protein in the absence of other viral proteins. This ectopic expression provides relevant information, although does not acknowledge the complex interplay of proteins during virus infection. A critical review of the literature may be helpful to interpret apparent discrepancies in the conclusions obtained by different experimental approaches. This review summarizes the current knowledge on human CoV accessory proteins, with an emphasis on their contribution to virus-host interactions and pathogenesis. This knowledge may help the search for antiviral drugs and vaccine development, still needed for some highly pathogenic human CoVs.

## Introduction

Since the identification of the first human coronavirus (HCoV) in the 1960s (Kendall et al., 1962), seven HCoVs have been described. Four of them, HCoV-229E, HCoV-NL63, HCoV-OC43 and HCoV-HKU1, are low pathogenic and cause mild symptoms like a common cold, while three of them, SARS-CoV, MERS-CoV and SARS-CoV-2, are potentially deadly for humans.

HCoVs belong to the *Coronaviridae* family, included in the *Nidovirales* order (Zhou et al., 2021). The *Orthocoronavirinae* subfamily comprises viruses that infect mammals and birds, which are classified into four genera: *alphacoronavirus* and *betacoronavirus*, including viruses that infect humans and other mammals, *gammacoronavirus*, which comprises viruses infecting birds, and *deltacoronavirus*, containing viruses that infect mammals and birds. HCoV-NL63 and HCoV-229E belong to the *alphacoronavirus* genus, while HCoV-OC43, HCoV-HKU1, SARS-CoV, MERS-CoV and SARS-CoV-2 belong to the *betacoronavirus* genus. This last genus has been recently subdivided into three subgenera: *Embecovirus* (HCoV-OC43 and HCoV-229E), *Sarbecovirus* (SARS-CoV and SARS-CoV-2) and *Merbecovirus* (MERS-CoV) (Gorbalenya et al., 2020; Kirtipal et al., 2020). The recent emergence of three highly pathogenic HCoVs during the 21th century (2002, 2012 and 2019, respectively) and the ongoing COVID-19 pandemic caused by SARS-CoV-2 have generated extensive interest in understanding the role of CoV proteins in pathogenesis.

**Figure 1:**
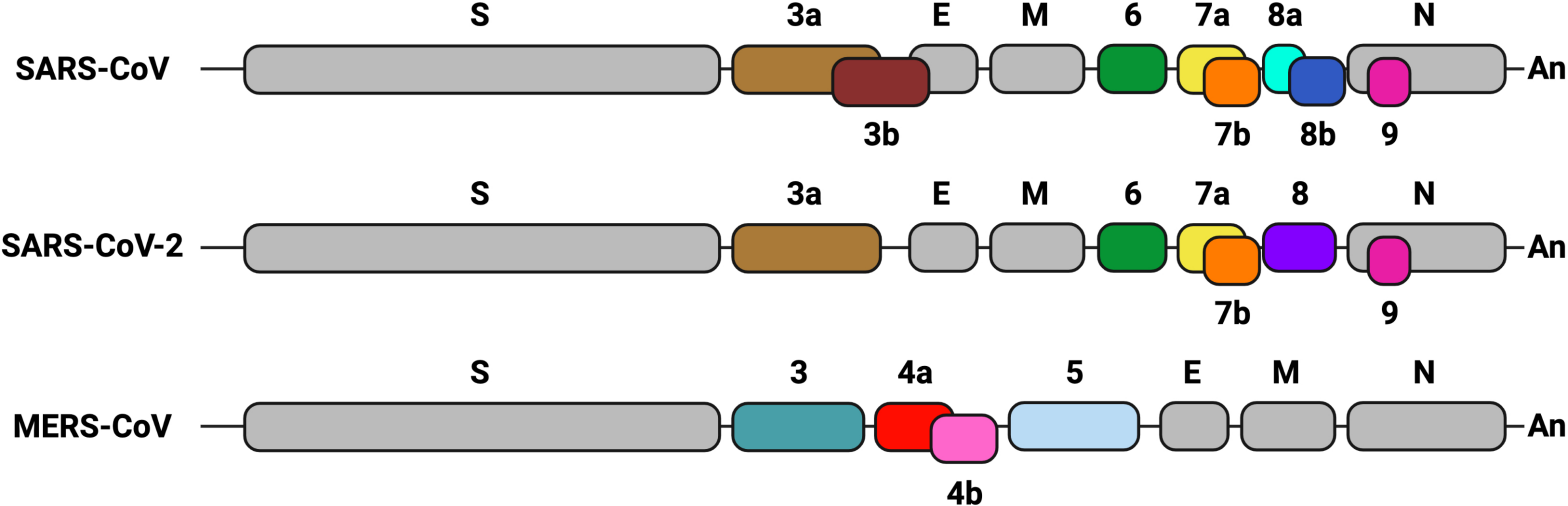
Genetic organization of the genome 3’ end in highly pathogenic human CoVs. Structural and accessory genes are shown. Gray boxes indicate structural genes: spike (S), envelope (E), membrane (M) and nucleocapsid (N). Accessory genes are represented in different colors. Their names are indicated as numbers above or below the boxes. Sequence conservation of accessory genes is indicated with the same color. The Poly A sequence (An) is represented at the 3´end of each CoV genome. Created with BioRender.com.

CoVs possess a positive-sense single-strand RNA genome (~ 30 kb). The 5’-proximal two-thirds of the genome comprise two open reading frames (ORF1a and ORF1b), encoding two polyproteins that are processed by viral proteases into 16 non-structural proteins involved in viral continuous RNA synthesis or replication and discontinuous transcription (Sola et al., 2015). The 3’ end of the genome contains genes that encode structural and accessory proteins. Four major structural proteins are homologous among all CoVs: spike (S), envelope (E), membrane (M) and nucleocapsid (N). Accessory genes, which encode structural and non-structural proteins, are variable in number, function and genome location among different CoV genera (Fang et al., 2021) (**Figure 1**). Accessory genes are interspersed among, or even overlapping, the structural genes. Accessory proteins are not essential for virus replication in cell cultures, although some of them may contribute to virus growth to some extent. However, they are involved in virus virulence *in vivo* by modulating cellular processes such as innate immunity, including the interferon (IFN) and proinflammatory responses, autophagy, cell cycle, apoptosis or stress pathways. In fact, alterations in these cell processes are often associated with the pathogenesis of deadly human CoVs, to which some accessory proteins contribute as virulence factors.

Understanding pathogenic virus-host interactions mediated by human deadly CoV accessory proteins is important to design antivirals that limit the severity of viral pathology. Monitoring mutations that these proteins undergo during viral evolution is essential to study changes in their interactions with cellular proteins. Moreover, deletion of accessory genes that behave as virulence factors from the viral genome provides a strategy to attenuate the virus for the development of vaccine candidates. Although other reviews have addressed the topic of SARS-CoV (Liu et al., 2014), MERS-CoV (Li et al., 2019) or SARS-CoV-2 (Redondo et al., 2021) accessory genes separately, this review is intended to provide a comparative description of the biological functions of accessory proteins from all three deadly human coronaviruses, including SARS-CoV and SARS-CoV-2 conserved proteins, with special emphasis on their contribution to pathogenesis. There is abundant literature describing the biological effects of CoV accessory proteins, but a significant proportion of these studies was performed by overexpressing a single protein. In the absence of other viral proteins, this ectopic expression may lead to non-physiological conditions with respect to subcellular localization, protein-protein interactions, or titration of host factors by the overexpressed protein, which could explain some controversial results. When possible, we will prioritize studies performed in the context of infection, which provide more physiological results.

## Interferon and proinflammatory antiviral responses

**Figure 2:**
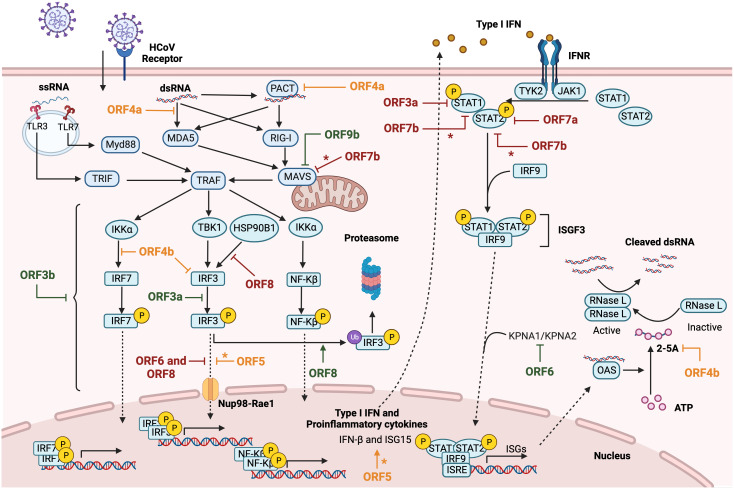
Modulation of IFN response by HCoVs accessory proteins. Diagram representing type I IFN and proinflammatory cytokines induction after cellular recognizing of HCoV RNA species by PRRs. After viral RNA sensing, MAVS-mediated nuclear transcription factor NF-Kβ and IRFs activation induce IFN and proinflammatory cytokines expression. The canonical autocrine or paracrine type I IFN signaling pathway triggers ISGs expression through ISGF3 complex activation. SARS-CoV accessory proteins are represented in green, MERS-CoV in orange and SARS-CoV-2 in red. The asterisk (*) indicates inconclusive results. Figure created with BioRender.com.

**Figure 3:**
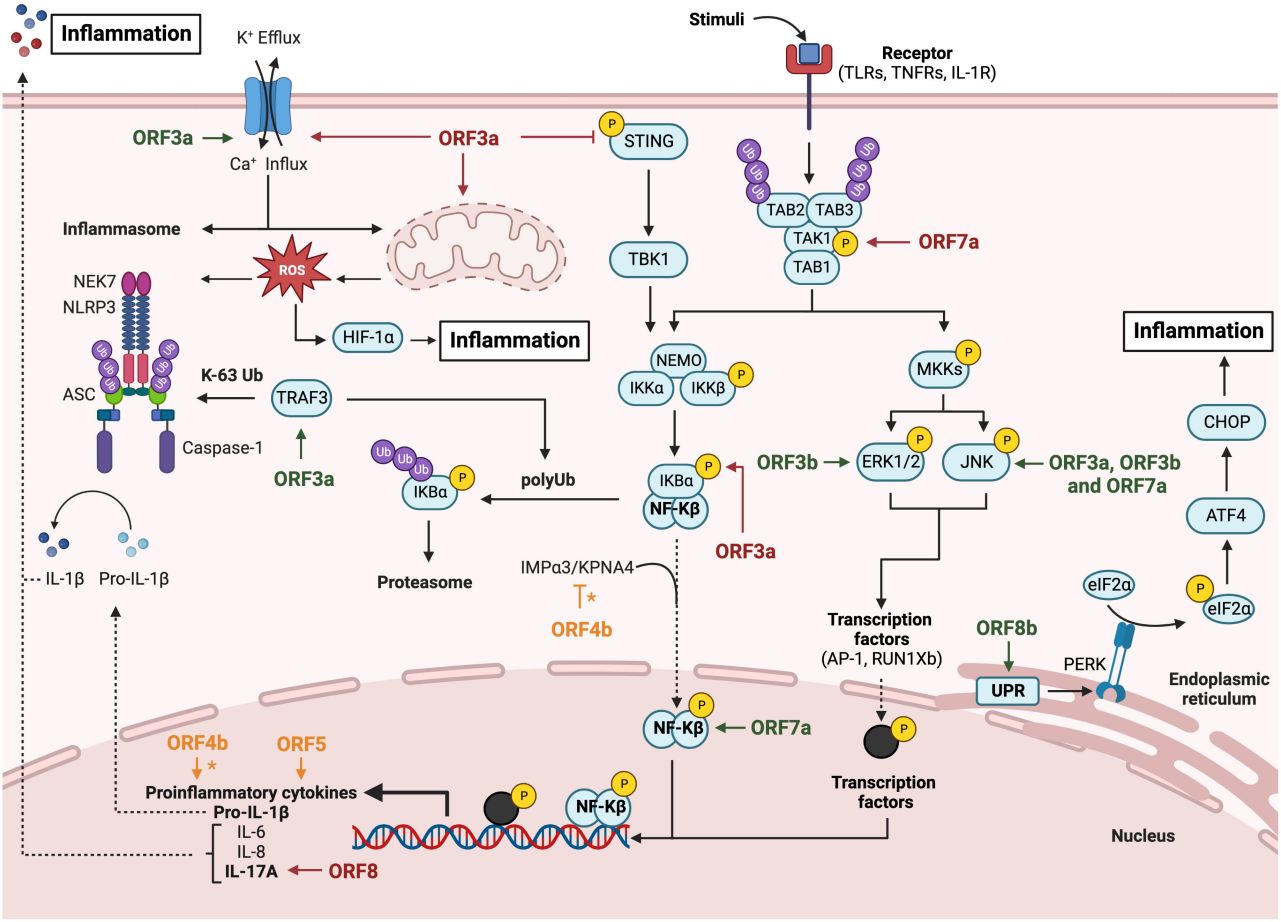
Modulation of inflammatory response pathway by HCoVs accessory proteins. Diagram representing the activation of the NLRP3 inflammasome pathway, the canonical NF-Kβ pathway and the canonical MAPKs pathway. The inflammatory inductors HIF-1α and CHOP activation are also represented via mitochondrial dysregulation and unfolded proteins response, respectively. SARS-CoV accessory proteins are represented in green, MERS-CoV in orange and SARS-CoV-2 in red. The asterisk (*) indicates inconclusive results. Figure created with BioRender.com.

The innate immune response represents the first line of defense against pathogens in vertebrates. Innate immunity includes a variety of cellular processes working coordinately to limit viral infection, including entry, translation and replication (Lokugamage et al., 2020). Innate immune response also contributes to identify and remove infected cells and to promote the adaptive immunity (Evans and Ozer, 1987). An essential component of innate immunity are cytokines, soluble factors that mediate the communication between cells and regulate different signaling pathways, such as the interferon (IFN) and pro-inflammatory responses. Type I IFN (IFNα and IFNβ) drives an antiviral state in non-immune cells and regulates antiviral responses to inhibit viral replication in infected cells (Lokugamage et al., 2020). IFN I activates the adaptive immune response by enhancing antigen presentation and promoting the action of T and B cells (Evans and Ozer, 1987; McNab et al., 2015). Pathogen-associated molecular patterns (PAMPs) or danger-associated molecular patterns (DAMPS) are recognized by pattern-recognition receptors (PRRs) of immune and non-immune cells, expressed in both the cell membrane (toll-like receptors or TLR) and the cytoplasm (retinoic acid associated gene-I or RIG-I; melanoma differentiation-associated gene 5 or MDA-5; and cytosolic DNA sensors or CDSs). It was described that long dsRNAs generated during CoV replication were preferentially detected by MDA5. The presence of a cap structure on CoV mRNAs (Park et al., 2022; Yan et al., 2022), similarly to eukaryotic mRNAs, protects from exonucleases and promotes mRNA translation and evasion of the host immune response. In contrast, RIG-I is activated by short uncapped dsRNAs containing 5′-PPP or 5′-PP groups, usually found in the genomes of negative-strand RNA viruses (Roth-Cross et al., 2008; Sampaio et al., 2021). Upon viral sensing, PRRs initiate signalling cascades that converge on activation and subsequent nuclear localization of different families of transcription factors, including interferon regulatory factors (IRFs) (Mogensen TH., 2018) and NF-κB, which leads to pro-inflammatory responses (Chen et al., 2018). The IFN expression pathway is induced by IFN regulatory transcription factors 3 (IRF3) and 7 (IRF7) (Mogensen TH., 2018), whose activation depends on the phosphorylation induced by IκB kinases, specifically, the TANK-binding kinase 1 (TBK1) (Moser et al., 2015), resulting in transcription of IFN-β or IFNα genes and production of the first wave of IFN-I (**Figure 2**). IFNs activate a signal transduction cascade when interacting with their receptors (IFNAR) in an autocrine and paracrine manner. This interaction activates the JAK/STAT pathway that phosphorylates the signal transducer and activation of transcription 1 and 2 (STAT1 and STAT2), which interact with IRF9 to form the ISGF3 complex. Binding of ISGF3 to the Interferon-Stimulated Response Element (ISRE) present in many promoters initiates the expression of hundreds of interferon-stimulated genes (ISGs), which exert antiviral activities and recruit immune cells to facilitate viral clearance (Basler and Garcia-Sastre, 2002; Schneider et al., 2014; Schoggins, 2019). The relevance of the IFN system is emphasized by the fact that most viruses have evolved different mechanisms that antagonize IFN production or signaling to enhance IFN resistance (Garcia-Sastre et al., 1998; Basler et al., 2000; Park et al., 2003; Garcia-Sastre, 2017). NF-κB, activator protein-1 (AP-1), or JAK-STAT proinflammatory pathways lead to the production of inflammatory cytokines and chemokines for the recruitment of inflammatory cells, as well as for the induction of cell death to clear infected cells (Koyama et al., 2008; Takeuchi and Akira, 2010; Diamond and Kanneganti, 2022) (**Figure 3**). A crosstalk between innate immune pathways, such as IFN and inflammation, is involved in the complex regulation of antiviral activity. For instance, the activation of the NF-kB pathways not only induces the expression of pro-inflammatory cytokines, but also facilitates transcription of type I IFN early after infection (Basagoudanavar et al., 2011).

## Interferon antagonism by deadly HCoV accessory proteins

Highly pathogenic HCoVs have developed strategies to evade the host IFN I response (Xue et al., 2021) in order to successfully infect their hosts (**Figure 2**). Inhibition of IFN production by individual accessory proteins from deadly HCoVs overexpressed in cell cultures has been extensively studied. SARS-CoV ORF3a inhibited phosphorylation of IRF-3 in HEK-293T, preventing the transcription of IFN-β gene (Kopecky-Bromberg et al., 2007). Other study described that overexpression of SARS-CoV ORF3b in HEK-293T cells inhibits type I IFN induction triggered by the overexpression of RIG-I or the mitochondrial antiviral signaling protein (MAVS), suggesting that 3b inhibits the IFN pathway at or downstream of the mitochondrial adaptor MAVS (Freundt et al., 2009). In addition, overexpressed SARS-CoV 8b and 8ab proteins induced degradation of IRF3 in a ubiquitin-proteasome-dependent manner, since IRF3 levels were partially restored in the presence of protease inhibitors (Wong et al., 2018). In agreement with this last result, the expression of 8b and 8ab SARS-CoV proteins by recombinant avian IBV viruses conferred a replication advantage to IBV in cells in which poly (I:C) induced the IFN response, suggesting a role for these proteins in the IFN antagonism. This effect was mediated by the down-regulation of IRF3 levels coupled to reduced IRF3 dimer formation.

SARS-CoV ORF6 regulates the IFN signaling, since infection with SARS-CoV blocks the nuclear trafficking of STAT1 in Vero cells transfected with STAT1-GFP, in contrast to deletion mutants lacking ORF6. A potential mechanism for this effect is that SARS-CoV ORF6 retains karyopherins, which are proteins involved in the transport of molecules between the cytoplasm and the nucleus, at the ER/Golgi membrane. During SARS-CoV infection, karyopherin 2 (KPNA2) in complex with KPNB1 were retained at the ER/Golgi, while KPNA2 was detected in the nucleus in SARS-CoV-Δ6 infection (Frieman et al., 2007). However, these results should be interpreted with caution because they were obtained in Vero cells, which are deficient in type I IFN production (Mosca and Pitha, 1986; Prescott et al., 2010).

Another SARS-CoV accessory protein described to inhibit MAVS is ORF9b. Overexpression of SARS-CoV 9b inhibited MAVS and reduced MAVS dependent-IRF3 phosphorylation. Moreover, it reduced in HEK-293 cells the levels of dynamin-like protein (DRP1), a host protein involved in mitochondrial fission, via a mechanism sensitive to proteasome inhibition. Lower DRP1 levels impaired the MAVS-induced IFN-β response (Shi et al., 2014).

SARS-CoV-2 infection induces in humans low levels of type I interferon (Blanco-Melo et al., 2020; Hojyo et al., 2020; Sa Ribero et al., 2020), suggesting that this CoV also encodes IFN antagonists. Similarly to SARS-CoV ORF3a, the overexpression of SARS-CoV-2 ORF3a in HEK-293T also inhibits IFN response, as expected from the high sequence conservation of 3a. In the case of SARS-CoV-2, two possible mechanisms have been suggested to inhibit the JAK/STAT signalling by ORF3, the suppression of STAT1 phosphorylation and nuclear translocation (Xia et al., 2020) or the upregulation of cytokine signalling 1 (SOCS1), a negative regulator of cytokine signalling (Wang et al., 2021). In agreement with these results, infection of human lung cells A549-hACE2 with SARS-CoV-2-Δ3a increased the expression levels of several ISGs (IFITM1, ISG56, OAS1 and PKR) as compared to the infection with the WT virus (Liu Y. et al., 2022).

The overexpression of all individual genes in HEK-293T cells transfected with an IFN-promoter reporter plasmid showed that ORF6 is the most potent IFN inhibitor of SARS-CoV-2 (Yuen et al., 2020). In this system, ORF6 directly binds the nuclear pore component Nup98-Rae1 and inhibits the nuclear translocation of activated STAT1 and IRF3 transcription factors, similarly to the function previously proposed for ORF6 of SARS-CoV (Miorin et al., 2020). The crystal structure of SARS-CoV-2 ORF6 C-termini in complex with the Rae1–Nup98 heterodimer showed that ORF6 occupies the mRNA-binding groove, similarly to other viral proteins, indicating that this complex is a common target for different viruses to impair the nuclear mRNA export pathway (Li et al., 2021).

Using recombinant SARS-CoV mutants with the ORF6 deleted (rSARS-CoV-ΔORF6) or replaced by SARS-CoV-2 ORF6 (rSARS-CoV_ORF6-SARS-2_), the IFN signaling pathway was evaluated in Vero E6 cells treated with IFN-I. Deletion of ORF6 significantly increased induction of IFN-stimulated genes *MX1* and ISG56, indicating that ORF6 was an antagonist of IFN-I signaling in the context of infection. rSARS-CoV_ORF6-SARS-2_ suppressed induction of *MX1* and ISG56 to a lesser extent than the wild-type SARS-CoV, suggesting that SARS-CoV-2 ORF6 interferes less efficiently with human IFN signaling than SARS-CoV ORF6. Since both genes are homologous, differences at charged amino acids at positions 51 and 56 may be responsible for the differential phenotype (Schroeder et al., 2021).

ORF7a is a type I transmembrane protein highly conserved in SARS-CoV and SARS-CoV-2. SARS-CoV-2 ORF7a also antagonized IFN-I signaling in overexpression experiments in HEK293T and A549 cells, by blocking STAT2 phosphorylation (Xia et al., 2020; Garcia-Garcia et al., 2022). Another study showed that ubiquitination of ORF7a at residue K119 was required for IFN antagonism, as overexpression of an ubiquitination-defective ORF7a in HEK293T failed to inhibit ISG production (Cao et al., 2021). These results are in agreement with the fact that a SARS-CoV-2 isolated from patients containing a 7-nucleotide deletion (nt 301-307) in ORF7a, that truncates the C-terminal half of the protein, is deficient in antagonizing the IFN response. This isolate induced elevated type I IFN responses in HEK293T-hACE2 cells, including high levels of the toll-like receptor 7 sensor (TLR7), signal transducers (MYD88, OAS2), transcriptional regulators (IRF3, IRF5), and restriction factors (GBP1, IFITM3, MX1), as compared to infection with the WT virus (Nemudryi et al., 2021). Another function proposed for ORF7a protein is the inhibition of tetherin BST-2, a host ISG that inhibits the release of virions and limits viral spread (Taylor et al., 2015; Martin-Sancho et al., 2021).

The role of SARS-CoV-2 ORF7b in the IFN pathway is not clear yet, since discrepant results were obtained by overexpressing the protein in cell cultures. The most conclusive studies showed that overexpression of ORF7b inhibited the IFN production. In HEK 293T, the ORF7b inhibited the MAVS-induced IFN-I and IFN-III production, but not the interferon signaling (Shemesh et al., 2021). In line with this last study, overexpression of ORF7b in A549 cells did not inhibit the expression of ISGs like OASL, IFIT1 and IFIT2 (Garcia-Garcia et al., 2022). Additionally, a truncated ORF7b with a 382-nucleotide deletion (Δ382), derived from a SARS-CoV-2 natural isolate identified in Singapore and Taiwan in 2020 and associated with mild disease, lost the ability to suppress MAVS-induced type I IFN production (Shemesh et al., 2021).

SARS-CoV-2 ORF8 ectopically expressed in cell lines has been described as an antagonist of interferon production by decreasing the nuclear translocation of IRF3 (Li et al., 2020; Geng et al., 2021; Rashid et al., 2021a; Chen et al., 2022). The W45L mutation present in ORF8 of a natural isolate may help the virus to evade the immune response by increasing its binding affinity to IRF3, according to molecular docking analysis (Rashid et al., 2021b). Alternatively, the interaction of ORF8 and HSP90B1, a molecular chaperone that interacts with IRF3, observed after overexpression of ORF8 in HEK-293T cells, could explain the IFN antagonist activity. This hypothesis is supported by the observation that HSP90B1 knocking-down significantly decreased activation of IFN-I pathway by polyI:C (Chen et al., 2022). Unexpectedly, infection with rSARS-CoV-2-Δ8 of K18-hACE2 mice induced in the lungs higher levels of IFN-γ than rSARS-CoV-2-WT, although no difference in IFN-α levels was detected, indicating that ORF8 might have a limited impact on the IFN-I response *in vivo* (Silvas et al., 2021), in contrast to the results observed in overexpression experiments.

MERS-CoV-WT infection did not significantly induce either IFNβ or pro-inflammatory cytokines in Huh-7 and Calu-3 cells (Canton et al., 2018). In contrast, infection of MRC5 cells activated a potent innate immune response (Bello-Perez et al., 2022), suggesting cell type-specific differences in the IFN response. Interestingly, infection of knock-in mice expressing the human virus receptor (KI-hDPP4) induced moderate IFN-β levels. Therefore, deletion of accessory genes from the MERS-CoV genome is expected to have different effects on the host response depending on the experimental system.

MERS-CoV accessory genes 3, 4a, 4b and 5 are highly divergent in sequence to SARS-CoV accessory genes. MERS-CoV 4a protein includes a dsRNA binding domain (DRBD) highly conserved in host proteins such as protein kinase R (PKR) or the protein activator of PKR (PACT) (Siu et al., 2014). Stress granules (SGs) are dynamic cytoplasmic condensates of RNAs and proteins including mRNAs associated with stalled ribosome complexes and other RNA-binding proteins. SGs are proposed to contribute to the post-transcriptional regulation of gene expression under stress conditions and to exert specific antiviral functions, by providing a platform for interactions of proteins from innate immune signaling pathways (Wen et al., 2014). Activation of PERK/PKR either by ER-stress or dsRNA leads to eIF-2α phosphorylation and translation inhibition, which ultimately promotes SG formation (Shi et al., 2022). PKR is an IFN stimulated gene, while SGs are proposed to serve to the regulation of innate immune responses, thus illustrating the complex interplay of antiviral signaling pathways.

The overexpression of ORF4a in HeLa cells inhibited PKR activation, thus preventing stress granule formation (Rabouw et al., 2016). Accordingly, a recombinant MERS-CoV-Δ4ab lacking ORF4a and 4b induced SG formation in susceptible HeLa-hDPP4 cells, but not in Vero cells (Nakagawa et al., 2018), which are defective for IFN production, suggesting a relation between SGs and IFN response. In fact, ORF4a overexpression in HEK293T cells suggested a role in the antagonist of IFN (Niemeyer et al., 2013; Siu et al., 2014; Rabouw et al., 2016) by two possible mechanisms. ORF4a may inhibit the MDA5-induced IFN response by directly interacting with viral dsRNA via the ORF4a dsRNA binding domain (DRBD) (Niemeyer et al., 2013). A second model proposes that ORF4a interacts with PACT in an RNA-dependent manner inhibiting PACT-induced activation of RIG-I and MDA5. MERS-CoV 4a protein might inhibit PACT, RIG-I and MDA5 by competing for dsRNA binding (Siu et al., 2014).

In the context of infection, ORF4a antagonizes IFN production and signaling, as A549^DPP4^ cells infected with a recombinant MERS-CoV-Δ4a showed increased mRNA expression levels of interferon lambda (IFNL1) and ISGs OAS2 and IFIT1 (Comar et al., 2019; Comar et al., 2022). Unexpectedly, the specific deletion of ORF4a from MERS-CoV genome did not result in robust activation of either PKR and SG formation (Rabouw et al., 2016) or the IFN and oligoadenylate synthetase/ribonuclease L (OAS-RNase L) pathways, as compared to WT virus (Comar et al., 2019; Comar et al., 2022). These results suggest that other viral proteins present in MERS-CoV-Δ4a are inhibiting the host antiviral response during infection.

MERS-CoV 4b protein includes a nuclear localization signal and a phosphodiesterase (PDE) domain (Thornbrough et al., 2016; Canton et al., 2018). In Huh-7 and Calu-3 cells, deletion of ORF4b from the viral genome increased the induction of IFN- β. In contrast, in MRC-5 cells and in susceptible mice, 4b deletion reduced the IFN-β response and attenuated the virus (Canton et al., 2018; Bello-Perez et al., 2022). These differences underline the need to find alternative, more physiological experimental systems to study the host response induced by MERS-CoV. Differences in cell lines and times post infection, which may also be related to differences in viral titers, in the different studies might justify the diverse contribution of 4b protein to the innate immune response in mice and cell cultures.

MERS-CoV 4b protein phosphodiesterase activity antagonizes the OAS-RNase L pathway (Thornbrough et al., 2016) by degrading 2’,5’-oligoadenylates (2-5A) required to activate RNase L, an antiviral system that cleaves viral and host single-stranded RNA and contributes to the control of replication and early spread of viruses (Choi et al., 2015). Infection of Calu-3 cells with the recombinant virus MERS-CoV-Δ4b did not completely restored rRNA degradation by RNase L, suggesting that MERS-CoV may include additional mechanisms of RNase L antagonism (Thornbrough et al., 2016). In agreement with these results, individual deletion of ORF4a and ORF4b had minor effects on RNaseL activity in A549^DPP4^ cells (Comar et al., 2022). However, infection with double mutant viruses with an inactivated endoribonuclease (EndoU) along with deletion of ORF4a or inactivation of ORF4b PDE domain strongly inactivated dsRNA-induced innate immune pathways (Comar et al., 2022). Deletion of ORF4b in MERS-CoV reduced the IFN response in MRC5 lung cells and also in hDPP4-KI mice (Bello-Perez et al., 2022) at 4 and 6 dpi, as compared to the WT virus, suggesting that ORF4b was not an IFN antagonist at these time points. Since the IFN response in CoV infection varies with time, it cannot be excluded that ORF4b antagonizes IFN-β production at earlier times post infection (Bello-Perez et al., 2022).

MERS-CoV gene 5 is highly conserved in viruses isolated from humans and camels. However, virus adaptation to cell culture or mouse lungs led to mutations and deletions that prevented full-length protein expression, leading to a truncated protein that contains a 17-nucleotide (nt) deletion and a stop codon at position 108 (Gutierrez-Alvarez et al., 2021). There are controversial results published about the role of the ORF5 in the IFN system. ORF5 antagonized interferon in over-expression experiments in 293T cells and inhibited the nuclear translocation of the interferon transcription factor IRF-3. In line with these results, in cells infected with Sendai virus, a potent inducer of IFN, the overexpression of ORF5 led to the accumulation of IRF3 in the cytoplasm (Yang et al., 2013). In contrast, the deletion of ORF5 from MERS-CoV (rMERS-CoV-Δ5) led to a decreased IFN response both in hDPP4-KI mice lungs and in MRC-5 cells, as the mRNA levels of IFN-β and ISG15 were significantly reduced as compared to the parental rMERS-CoV virus expressing the truncated protein (Gutierrez-Alvarez et al., 2021). In addition, another study suggested no specific role of ORF5 in the IFN antagonism, since the replacement of ORF5 by a fluorescent gene failed to induce in Calu 3 cells robust type I and III IFN responses, at different times post-infection (Menachery et al., 2017). Differences among studies regarding the relevance of gene 5 to the IFN response highlight the importance of using physiological experimental systems that recapitulate the complexity of virus-host interactions in the context of viral infection at the organism level.

A relationship between interferon antagonism and viral pathogenesis has been established (Thornbrough et al., 2016). Viruses may inhibit the induction of the IFN response by blocking the IFN signaling, or by interfering with the antiviral activities induced by IFN. In highly pathogenic coronaviruses SARS-CoV and MERS-CoV, a late or prolonged IFN-β response increase virulence and decrease survival, while early IFN-ß production was associated with protection (Channappanavar et al., 2019).

## Interference of deadly HCoV accessory proteins with the inflammatory response

Highly pathogenic human CoVs induce exacerbated inflammatory processes, which are a main determinant of pathogenesis (Lowery et al., 2021). A limited and delayed IFN-I and IFN-III response results in exacerbated proinflammatory cytokine production and in extensive cellular infiltrates in the respiratory tract, resulting in lung pathology. High levels of inflammatory markers, such as increased neutrophil-lymphocyte blood ratio or cytokine-chemokine serum levels have been associated with higher risk of severe disease in SARS-CoV-2 infections (Hu et al., 2021). It is known that SARS-CoV, MERS-CoV and SARS-CoV-2 infections dysregulate inflammation leading to cytokine storm and acute respiratory distress syndrome (ARDS), which are described as hyperinflammatory pathologies (Chien et al., 2006; Kim et al., 2016; Liu T. et al., 2021). Among HCoVs genes, accessory genes have been proposed to interfere with the innate immune response and particularly, with the inflammatory pathway (**Figure 3**).

Several SARS-CoV accessory proteins interfere with inflammatory processes. ORF3a and 8a are viroporins, viral transmembrane proteins with ion channel activity. ORF3a has permeability to calcium, potassium and sodium ions, which has effects on cellular homeostasis. Additionally, protein 3a has a PDZ-binding motif (PBM), which can potentially participate in protein-protein interactions with over 400 cellular proteins that contain PDZ domains. SARS-CoV 3a protein was required for efficient SARS-CoV replication and virulence in mice, whereas viroporin 8a had only a minor impact on these activities (Castano-Rodriguez et al., 2018). A recombinant mouse adapted SARS-CoV-Δ3a-MA30 was attenuated in BALC/c mice, indicating that ORF3a was a virulence factor. However, individual mutants of ORF3a ion channel or PBM domains were not attenuated, suggesting that, separately, these activities did not play a key role in pathogenesis (Castano-Rodriguez et al., 2018).

In HeLa cells overexpressing SARS-CoV ORF3a, NLRP3 inflammasome was activated in an ion channel-activity dependent manner, leading to IL-1β secretion. Dysregulation of potassium flux by protein 3a, which alters cell homeostasis, and the production of reactive oxygen species from damaged mitochondria were required for NLRP3 activation (Chen et al., 2019). Another mechanism that may contribute to NLRP3 inflammasome activation is the canonical NF-kB signaling pathway. HEK293T and A549 cells overexpressing ORF3a showed activation of NF‐κB and JNK1, which led to increased expression of IL-8 and RANTES (Kanzawa et al., 2006). Activation of the canonical NF-κB signalling pathway involves the participation of TNF receptor-associated factor 3 (TRAF3) in the degradation of the IkBα inhibitor and the processing of NF-kB p105 into p50, which promotes IL-1β transcription. ORF3a has been described to bind the inflammasome adapter protein ASC promoting its polyubiquitination, a critical step required for NLRP3 inflammasome activation (Siu et al., 2019). In line with the overexpression results, deletion of 3a gene from the viral genome (SARS-CoV-Δ3a) confirmed that ORF3a is necessary for NLRP3 inflammasome activation and IL-1β production, although this effect was independent of the ion-channel activity (Siu et al., 2019).

Another SARS-CoV accessory protein related to the activation of inflammatory pathways is ORF3b. Overexpression of ORF3b in Huh7 cells led to the activation of two MAPKs, JNK and ERK, which increased the activity of the transcription factor AP-1, leading to enhanced expression of proinflammatory mediators like the monocyte chemoattractant protein-1 (MCP-1 also known as CCL2) (Varshney and Lal, 2011). A more extensive analysis of interactions of overexpressed ORF3b with host factors in Huh7 cells, using yeast two-hybrid and co-immunoprecipitation techniques, identified the transcription factor RUNX1b as a partner of 3b. Physical interaction between ORF3b and RUNX1b increased its transactivation potential via ERK phosphorylation, leading to enhanced transcription of the macrophage inflammatory protein MIP-1α and the proinflammatory cytokine IL2 (Varshney et al., 2012). Monocyte cells are abortively infected by SARS-CoV. However, it is speculated that the expression of 3b protein from the viral genome in infected monocytic cells might induce inflammatory responses and contribute to SARS-CoV pathogenesis.

SARS-CoV ORF7a is also described as a proinflammatory accessory protein by enhancing NF‐κB as well as JNK1 phosphorylation levels, which led to increased levels of proinflammatory cytokines IL-8 and RANTES in A549 cells overexpressing ORF7a (Kanzawa et al., 2006). In addition, SARS-CoV ORF8b has been described in overexpression systems as an insoluble accessory protein. Intracellular aggregates of ORF8b induced both endoplasmic reticulum and lysosomal stress leading to the accumulation and activation of the transcription factors CHOP and TFEB, both related to proinflammatory responses. This study also demonstrated that ORF8b activates NLRP3 inflammasome in macrophages and monocytes (Shi et al., 2019).

SARS-CoV-2 accessory proteins are also related to proinflammatory processes. As described for SARS-CoV, the highly conserved SARS-CoV-2 ORF3a activates the NLRP3 inflammasome (Xu et al., 2022). Overexpression of ORF3a in A549 cells induced potassium efflux, via ORF3a ion channel activity, which triggers the interaction of NLRP3 (**Figure 3**) with the activator kinase NEK7, leading to the recruitment of the inflammasome adapter protein ASC and pro-caspase 1 into the active inflammasome. In addition, ORF3a increased IL1-β levels due to NF‐κB induction via IκBα phosphorylation (Xu et al., 2022). Another study related ORF3a overexpression in HEK293T cells to the inhibition of the proinflammatory response by preventing cGAS-STING immune activation (Rui et al., 2021). This inhibition may be due to the direct interaction between ORF3a and STING, as suggested by colocalization and coimmunoprecipitation studies. In fact, it was shown that ORF3a inhibited NF‐κB induction by cGAS-STING antagonism (Rui et al., 2021). Furthermore, ORF3a overexpressed in HEK293T and Hela cells increased the levels of the transcription factor HIF-1α, by inducing mitochondrial damage and mitochondrial reactive oxygen species (Mito-ROS) signalling, ultimately promoting transcription of pro-inflammatory cytokines IL-6, and IL-1β (Tian et al., 2021).

SARS-CoV-2 ORF7a overexpressed in HeLa and A549 cells activated the NF‐κB-dependent proinflammatory response, as it was previously described for SARS-CoV ORF7a (Su CM. et al., 2021) (**Figure 3**). Similar results were reported in HEK293T cells, leading to IL8 and IP-10 production. This study suggested that ORF7a induces the NF‐κB pathway by binding and activating the ubiquitin-dependent kinase TAK1. ORF7a polyubiquitination, mediated by the E3 ubiquitin ligase RNF121, was required for NF‐κB pathway activation. Polyubiquitinated ORF7a may need the K63-linked polyubiquitin binding activity of TAK1 binding proteins TAB2 and TAB3 to activate TAK1, which leads to NF‐κB activation. In addition, binding of the NF-kB modulator NEMO to polyubiquitin was also needed for this activation (Nishitsuji et al., 2022).

The sequence identity between SARS-CoV and SARS-CoV-2 ORF8 is low (~26%) (Takatsuka et al., 2022), suggesting different roles in the immune response pathways. SARS-CoV-2 ORF8 acts as an IL17a mimetic by interacting with its receptor IL17RA, thereby activating this pathway (Lin et al., 2021; Wu et al., 2022). ORF8 overexpressed in HEK293T cells interacted with IL17a receptor (IL17RA and IL17RC), inducing a proinflammatory response. ORF8 amino acids Y42, E106, E171 and E176 played a critical role in this interaction (Wu et al., 2022). In another paper, adenoviruses expressing ORF8 rescued the IL17A phenotype in IL17A-deficient mice, suggesting that ORF8 mimics the pro-inflammatory function of IL17A (Lin et al., 2021).

The role of MERS-CoV accessory proteins on inflammatory pathways is cell type-dependent. MERS-CoV ORF4b has been related to NF‐κB inhibition, thus reducing inflammatory responses in cells (Matthews et al., 2014; Munasinghe et al., 2022). One study in which ORF4b was overexpressed in HEK293T cells treated with TNF-α, showed a modest (two-fold) reduction in expression of genes regulated by NF‐κB transcription factor (Matthews et al., 2014). Another study (Munasinghe et al., 2022) also described that ORF4b overexpression inhibited the NF‐κB pathway by binding the host nuclear import protein IMPα3 (also known as karyopherin-α4), via the 4b nuclear localization sequence (NLS), which inhibited NF‐κB nuclear translocation. Computational analysis showed the potential of ORF4b to competitively inhibit IMPα3 by binding the major NLS site of IMPα3, which is necessary for NF-KB p50 nuclear import (Munasinghe et al., 2022). The role of 4b protein in the pro-inflammatory response has also been demonstrated in the context of infection. Recombinant viruses, MERS-CoV-Δ4b and MERS-CoV-mNLS, in which the 4b gene was either deleted or its NLS inactivated by mutation, respectively, induced higher levels of NF‐κB-dependent pro-inflammatory cytokines IL6, IL8 or TNF-α in Huh7 and Calu-3 cells, as compared to the rSARS-CoV-WT. This result indicated that 4b protein inhibits the inflammatory response during infection and its nuclear localization is necessary for NF‐κB inhibition. The study described the interaction between ORF4b and karyopherin-α4 (KPNA4) in an NLS-dependent manner. KPNA4 is known to translocate the NF-κB protein complex from the cytoplasm into the nucleus. Binding of 4b to KPNA4 during infection inhibited its interaction with NF-κB-p65 subunit. Thereby, a model where 4b outcompetes NF-κB for KPNA4 binding and translocation into the nucleus was proposed as a mechanism of interference with the NF-κB-mediated response (Canton et al., 2018). *In vivo* experiments in the hDPP4-KI mouse model, using mouse-adapted recombinant viruses with identical ORF4b mutations, showed that 4b protein is a virulence factor, since MERS-CoV-Δ4b is completely attenuated, in contrast to MERS-CoV-WT, which causes 100% mortality. However, deletion of 4b significantly reduced the expression of pro-inflammatory cytokines and chemokines and the accumulation of cell infiltrates in the lungs of mice 3 and 6 dpi (Bello-Perez et al., 2022). The apparent discrepancy between the results of 4b protein during infection of cell lines or hDPP4-KI mice might be explained by the timing of the innate immune response. In cell lines, deletion of 4b increased the pro-inflammatory response at 24 hpi, while the reduced inflammation *in vivo* was detected at 3 and 6 dpi. Further studies would be required to confirm whether 4b deletion promoted *in vivo* an inflammatory response at earlier times with protective effects.

MERS-CoV ORF5 also dysregulates the inflammatory response. A recombinant mouse adapted MERS-CoV virus lacking ORF5 (rMERS-Δ5-MA15) induced delayed and dysregulated levels of proinflammatory cytokines in hDPP4-KI mice at 4 dpi. Similarly, lower levels of proinflammatory mediators were induced in MRC-5 cells at 18-24 hpi. This result suggests a role of ORF5 in inflammatory modulation. Interestingly, rMERS-Δ5-MA15 was more virulent in mice than WT, indicating a role of gene 5 in pathogenesis (Gutierrez-Alvarez et al., 2021).

## Apoptosis

**Figure 4:**
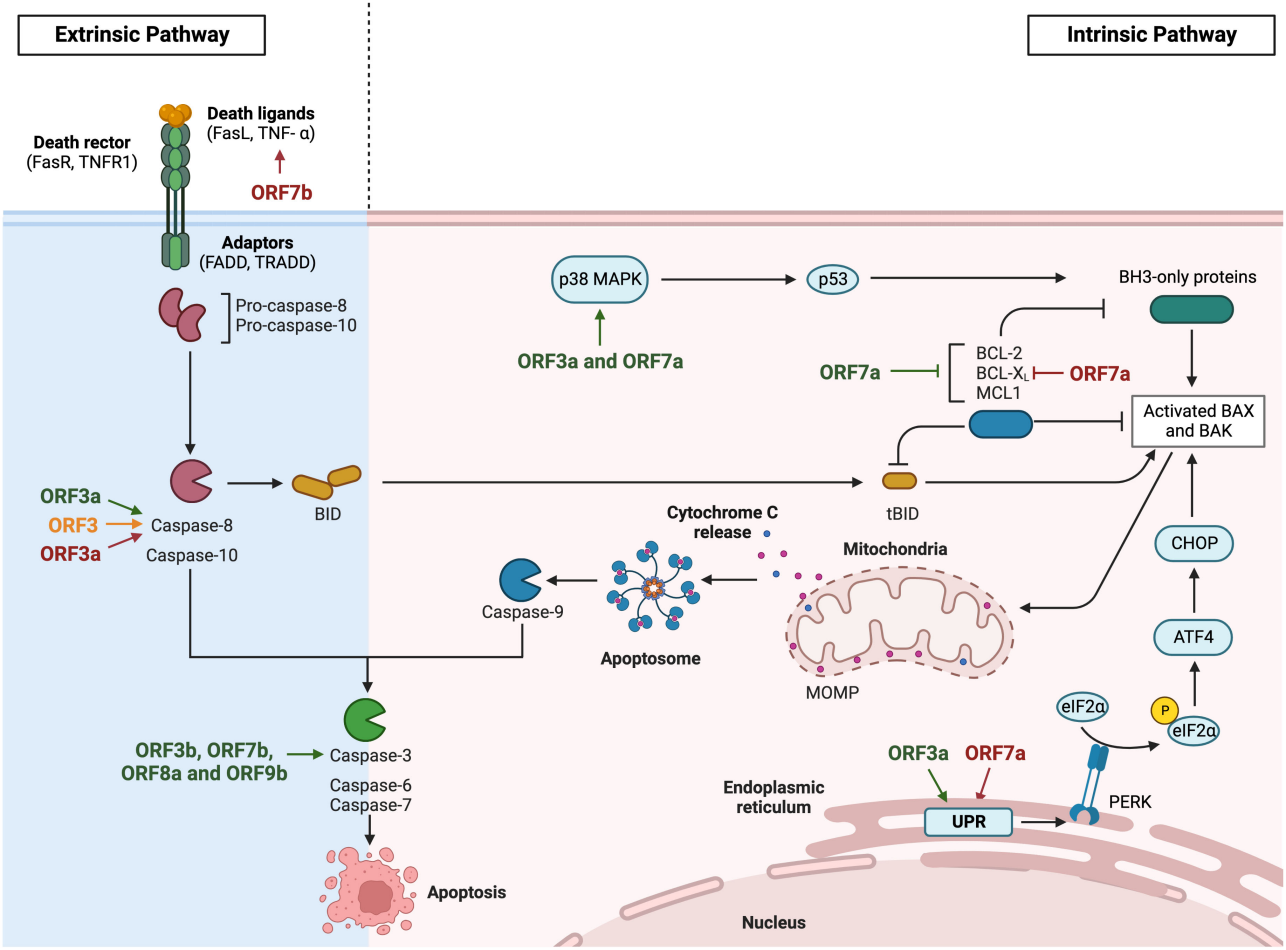
Modulation of apoptosis by HCoVs accessory proteins. Diagram representing the extrinsic and the intrinsic apoptosis pathway via death ligand recognition or viral induction respectively. The apoptosis inductor CHOP activation is represented via unfolded protein response. SARS-CoV accessory proteins are represented in green, MERS-CoV in orange and SARS-CoV-2 in red. Images created with BioRender.com.

Apoptosis is a programmed cell death characterized by cell cycle arrest and controlled phagocytosis of cellular components, previously released following cell fragmentation in vesicles known as apoptotic bodies (Xu et al., 2019). The induction of this pathway is dependent on the activation of caspases (**Figure 4**), which are cysteine-aspartic proteases. Initiator caspases (caspases 8 and 9) are activated by proapoptotic proteins and induce effector caspases (caspases 3, 6 and 7), which initiate the apoptosis pathway. Once initiated, apoptotic cell DNA is fragmented by nucleases, while nuclear and cytoskeleton proteins are degraded and cellular components are cleared by phagocytic cells. Apoptosis is induced by two different pathways. The intrinsic pathway is modulated by the B cell lymphoma 2 (Bcl2) family of proteins. These proteins control the mitochondrial outer membrane permeability (MOMP), either by increasing permeability by proapoptotic factors like BAX and BAK, or by decreasing it by antiapoptotic factors like Bcl2, Bcl-xL and Mcl-1. MOMP increases the release of cytochrome C from the mitochondria to the cytoplasm, which activates the initiator caspase 9 and finally, the effector caspase 3. The extrinsic pathway is modulated by the binding of death ligands, like FasL or TNF-α, to death receptors present on the cell surface. This pathway activates the initiator caspase 8, which can both induce the effector caspases or coactivate the intrinsic pathway by the activation of proapoptotic Bid protein (Elmore, 2007).

Premature cell death induced by apoptosis is a potent antiviral pathway to limit viral spread (Shen and Shenk, 1995). Alternatively, some viruses induce an uncontrolled apoptosis that increases pathogenesis. This is the case of deadly HCoVs, which have been related to apoptosis induction (Yan et al., 2004; Yeung et al., 2016; Liu Y. et al., 2021). Although little information is available in the context of infection, apoptosis activation (**Figure 4**) may contribute to pathogenesis.

Among SARS-CoV accessory proteins, ORF3a has been proposed as an apoptosis inductor (Law et al., 2005; Padhan et al., 2008; Chan et al., 2009; Minakshi et al., 2009; Freundt et al., 2010). Despite the mechanism remains unclear, overexpression of ORF3a in Vero E6 cells activated the extrinsic pathway through caspase-8 activation (Law et al., 2005). In addition, overexpressed ORF3a induced the intrinsic pathway via activation of both caspase-8 and caspase-9 in Huh7 (Padhan et al., 2008) and Vero E6 cells (Chan et al., 2009). ORF3a C133S mutation, which disrupts ORF3a homo-dimer/-tetramer formation, and subsequently its ion channel activity, resulted in the loss of apoptosis induction, suggesting that ORF3a ion channel activity is the main contributor to its activity (Chan et al., 2009). ORF3a probably also activates apoptosis via the unfolded protein response (UPR) pathway (Minakshi et al., 2009). Huh7 cells overexpressing ORF3a activated PERK-eIF2α pathway, which induced translation of the activating transcription factor 4 (ATF4) that subsequently promoted transcription of the C/EBP homologous protein (CHOP) (**Figure 4**). CHOP upregulated the transcription of BAK and BAX, which oligomerize and release apoptotic factors, such as cytochrome c and apoptosis-inducing factor (AIF), through mitochondria permeabilization, eventually causing cell death (Minakshi et al., 2009). According to overexpression results, Vero E6 cells infected with SARS-CoV-Δ3a showed lower cell death levels than WT infection, suggesting an implication of ORF3a in cell death pathways (Freundt et al., 2010).

The overexpression of SARS-CoV 7a, but not 3a or the viral structural proteins, N, M, and E, induced apoptosis via a caspase-dependent pathway in cell lines derived from different organs, including lung, kidney, and liver. These results indicate that apoptosis activation by overexpressed viral proteins could be cell type dependent (Tan et al., 2004). In addition, ORF7a overexpressed in HEK293T cells inhibited protein synthesis by inducing a stress response that activated the p38 MAPK pathway, which in turn, could induce the apoptosis pathway (Kopecky-Bromberg et al., 2006). In this line, a physical interaction between ORF7a and several prosurvival Bcl-2 family proteins like Bcl-xL, Bcl-2, Bcl-w, A1 and Mcl-1 was shown by coimmunoprecipitation in Vero E6 infected cells. ORF7a transmembrane domain was proposed as the major contributor to this interaction. This interaction with antiapoptotic, but not with proapoptotic proteins, may lead to apoptosis induction (Tan et al., 2007). Another possible mechanism for apoptosis activation by ORF7a is related to the interaction of 7a with Ap_4_A-hydrolase, which plays a role in maintaining cell homeostasis by cleaving diadenosine tetraphosphate (Ap_4_A) back into ATP and AMP. It was suggested that ORF7a may be downregulating Ap_4_A-hydrolase activity, leading to increased Ap_4_A levels (Vartanian et al., 2003), which have been previously related to apoptosis induction (Vasilenko et al., 2010). In contrast, one study in the context of infection showed similar apoptosis levels in Vero E6 cells infected with rSARS-CoV-ΔORF7ab or the WT virus. Therefore, despite overexpression of ORF7a or ORF7b in Vero E6 cells increased caspase-3 activation, these proteins did not significantly contributed to apoptosis in the context of infection, which would be induced by other viral proteins (Schaecher et al., 2007). This discrepancy may be due to an artifact related to overexpression. It is known that overexpression of proteins induces endoplasmic reticulum stress, leading to apoptosis indirectly (Szegezdi et al., 2006). Although the mechanisms were not explored, there are some papers relating caspase-3 activation with the presence of overexpressed SARS-CoV accessory proteins like ORF3b (Yuan et al., 2005; Khan et al., 2006), ORF8a (Chen et al., 2007) or ORF9b accumulation in the nucleus (Sharma et al., 2011).

Apoptosis induction has also been described in SARS-CoV-2 infections (Liu Y. et al., 2021), as observed in postmortem lung sections from COVID-19 patients and in lung tissues from a non-human primate model of SARS-CoV-2 infection. As previously reported for SARS-CoV, accessory proteins have been associated with apoptosis activation. Similar to SARS-CoV ORF3a, overexpression studies suggested SARS-CoV-2 ORF3a as a proapoptotic protein via the extrinsic pathway, by activating caspase-8, which led to Bid cleavage, cytochrome c release and ultimately, the activation of the intrinsic pathway. ORF3a C130/133S or Y160A mutants failed to induce significant apoptosis levels, suggesting the relevance of the transmembrane domain in this function (Ren et al., 2020). In addition, overexpressed ORF7a induced apoptosis in HEK293T cells, by interacting with Bcl-xL, which was retained in the endoplasmic reticulum inhibiting its antiapoptotic activity (Liu Z. et al., 2022). This interaction was dependent on ORF7a C-terminal positively charged Lys117 and Lys119 residues, present in the extracellular domain (Liu Z. et al., 2022). The endoplasmic reticulum accumulation of ORF7a induced ER stress via PERK-eIF2α pathway, which upregulated the transcription factor CHOP, leading to the activation of the intrinsic pathway of apoptosis via BAK/BAX induction and cytochrome c release (Liu Z. et al., 2022). Finally, SARS-CoV-2 ORF7b overexpressed in Vero E6 and HEK293T cells was also related to apoptosis activation via the extrinsic pathway. An increase in TNF-α, which binds type I TNF receptor (TNFR1) and promote caspase-8 activation was described (Yang et al., 2021).

ORF3 is the only accessory protein of MERS-CoV that is related to apoptosis induction. Apoptosis analysis in HEK293T, BEASB2, Calu-3 and A549 cells overexpressing ORF3 demonstrated that ORF3 activates caspase-8 and caspase-3, but not BAX or Bcl-2 proteins, suggesting an effect in the apoptosis extrinsic pathway. It has been described that ORF3 was degraded via the ubiquitin-proteasome system, which limits the apoptosis induction. This polyubiquitination was performed by HUWE1, an E3 ligase that physically interacted with ORF3, thus preventing an exacerbated apoptosis activation (Zhou et al., 2022).

## Regulation of antigen presentation

Viruses present a variety of mechanisms to evade the host immune system. One of them is the expression of proteins with immunoglobulin-like (Ig-like) domains that may interfere with multiple functions, including antigen presentation, cell-cell recognition and signal transduction in the immune system (Gewurz et al., 2001; Fu et al., 2011). SARS-CoV-2 ORF8 contains an Ig-like domain, suggesting its role in mediating macromolecular interactions in immune responses (Chen et al., 2021). Cytotoxic T lymphocytes (CTL) recognition of infected cells is crucial for viral clearance. Higher levels of the cytokines induced by T cells (IFN-γ, TNF-α, IL-2, and IL-5) were detected in patients infected with the attenuated isolate SARS-CoV-2-Δ382, in which a 382 nt deletion truncates 7b and abolishes ORF8 expression, suggesting improved T cell recognition of infected cells in the absence of ORF8 (Young et al., 2020). SARS-CoV-2 infection led to MHC-I downregulation in infected hACE2-HEK293T cell, similarly to overexpressing of ORF8 in the absence of infection (Zhang et al., 2021a; Matsuoka et al., 2022). In these cells, the interaction of ORF8 with MHC-I and LC3-labeled autophagosomes was confirmed by co-localization and co-immunoprecipitation experiments (Zhang et al., 2021a). Inhibition of autophagy significantly restored the MHC-I surface expression, suggesting that downregulation of MHC-I by ORF8 involves the autophagy pathway (Zhang et al., 2021a). Lower expression of MHC-I can result in impairment of CTL-mediated lysis of SARS-CoV-2–infected cells, therefore reducing the antiviral cytotoxic immune response of the host, which may increase virus virulence. In fact, ORF8 protects target cells from CTL lysis, as SARS-CoV-2-specific CD8+ T cells isolated from recovered patients eliminated ORF8-expressing target cells with lower efficiency than control cells (Zhang et al., 2021a). ORF8 of SARS-CoV-2 delta variant includes deletions of Asp119 and Phe120 amino acids, which result in structural instability of ORF8 dimers. Docking analysis revealed reduced interaction of mutant ORF8 with MHC-I, as compared to the WT ORF8, suggesting that delta variant might not be able to bind efficiently the MHC-I complex, thus limiting MHC-I degradation via autophagy, which might result in a better immune response against this SARS-CoV-2 variant. In addition to ORF8, the overexpression of SARS-CoV-2 ORF7a in HEK-293 T cells reduced 5-fold the MHC-I levels on the cell surface. However, this down-regulation was largely maintained in cells infected with a recombinant SARS-CoV-2 in which the ORF7 was replaced by a reporter gene (Zhang F. et al., 2022), suggesting additional mechanisms of MHC-I downregulation, possibly by ORF8. SARS-CoV-2 ORF8 has also been proposed to modulate the recognition of viral antigens via antigen presenting monocytes, as immune cell-binding assays showed a stronger interaction of ORF8 with monocytes than with other immune cells (Chen et al., 2021).

## Histone mimic

CoVs use a variety of mechanisms to interfere with multiple host cell functions. One strategy is the interference with the epigenetic regulation of gene expression mediated by histone modification. SARS-CoV-2 ORF8 mimics the ARKS sequence present in histone H3, which is the most critical regulatory region for post-translational modifications (PTMs) (Kee et al., 2022). SARS-CoV-2 infection of A549 cells increased the levels of methylated histones H3K9me3 and H3K27me3 as compared with mock-infected cells (Kee et al., 2022). The complete deletion of ORF8, or just the ARKSAP motif, attenuated this effect, indicating that this motif was involved in histone modifications (Kee et al., 2022). In addition, infections with SARS-CoV-2-ΔORF8 or SARS-CoV-2-ΔARKSAP did not reduce the expression of the acetyltransferase histone KAT2A levels as in SARS-CoV-2-WT infection (Kee et al., 2022). The histone mimic function of ORF8 was also confirmed in post-mortem lung tissue samples from COVID19 patients, as infected cells showed stronger staining of methylated histone H3K9me3 than neighbouring cells or control tissues (Kee et al., 2022). The histone mimic function could contribute to the milder disease observed in patients infected with the SARS-CoV-2 isolate including a the ORF8 382-nt deletion. Remodeling of chromatin induced by acute viral infections can disrupt epigenetic regulation of gene expression with multiple effects on cellular processes, including the immune response (Kee et al., 2022). However, the implications of these chromatin modifications either in acute infection or in post-acute sequelae of SARS-CoV-2 are not well understood yet.

## Autophagy regulation

Autophagy is an intracellular catabolic transport route highly conserved among all eukaryotic cells. Among its many functions, this pathway plays a role in the defence against viruses. Autophagy sequesters viruses into double-membrane vesicles (autophagosomes) that subsequently fuse with lysosomes, forming the autolysosome, where viruses are degraded under acidic conditions (de Haan and Reggiori, 2008; Jackson, 2015; Choi et al., 2018; Bello-Perez et al., 2020). This process is orchestrated by Beclin 1, a scaffolding protein that regulates the lipid kinase Vps34 and interacts with ultraviolet irradiation resistance-associated gene (UVRAG) or the autophagy related gene 14 (Atg14) to form the PI3K3-C1 complex (Beclin 1-Vps34- Atg14) or PI3K3-C2 complex (Beclin1-Vsp34-UVRAG), which promote autophagosome formation or maturation, respectively (Qu et al., 2021). On the other hand, the autolysosome formation depends on the interaction between a specific SNARE (soluble N-ethylmaleimide-sensitive factor attachment protein receptor) called Syntaxin 17 (STX17) with the homotypic fusion and protein sorting (HOPS) complex (Jiang et al., 2014). The autophagy flux is measured by analyzing two markers: the conversion of microtubule-associated protein light chain 3 (LC3) to lipidated LC3-II, which correlates with the autophagosome formation, and the turnover of the autophagy substrate p62 (also known as SQSTM1). While the increase in LC3-II may reflect either increased autophagosome formation or accumulation due to decreased autolysosome degradation, p62 decrease indicates that autophagy is induced (Klionsky et al., 2021). Although many viruses inhibit autophagy to prevent their own degradation, others use this pathway to complete their morphogenesis. For this reason, autophagy could have either anti-viral or pro-viral effects (Prentice et al., 2004; Krejbich-Trotot et al., 2011; Tian et al., 2011).

Viral proteins, including accessory proteins, exploit different mechanisms to inhibit autophagy by blocking the progression of autophagosomes into autolysosomes. The incomplete autophagy induced by SARS-CoV-2 in infected HeLa-hACE2 and Vero E6 cells has been associated with ORF3a, because ORF3a overexpression inhibited the fusion between autophagosomes and lysosomes in multiple cell lines as HEK-293T (Hayn et al., 2021; Miao et al., 2021), A549 and HeLa-hACE2 (Zhang et al., 2021b). ORF3a directly sequesters the mediator of autophagy Vps39 on late endosomes and prevents its interaction with STX17, a membrane protein of autophagosomes that controls fusion with lysosomes, thereby blocking the autolysosome formation (Miao et al., 2021; Zhang et al., 2021b). In addition, ORF3a binds UVRAG, significantly reducing the interaction between autophagy factors UVRAG and Beclin 1, which may disrupt the formation of the PI3KC3-C2 complex involved in autophagosome maturation (Qu et al., 2021).

The overexpression of ORF7a causes the accumulation of autophagosomes in different cell lines, HEK-293T, Hela, Caco2 and Vero E6 (Koepke et al., 2021; Hou et al., 2022), as a consequence of the reduced acidity of lysosomes, which inhibited the autophagosomal degradation and prevented the autophagy flux (Koepke et al., 2021). Knock-down of ORF7a sgmRNA translation with specific short-hairpin RNAs (shRNAs) during infection decreased autophagy levels (Hou et al., 2022). Together, these results suggest that ORF7a initiates autophagy, but also limits the progression of the autophagy flux by activating caspase 3, which cleaves the SNAP29 membrane protein required for the fusion of autophagosomes and lysosomes (Hou et al., 2022). Consequently, ORF7a promotes viral replication by inhibiting autophagy progression.

Autophagy regulation was also shown to contribute to the virulence of CoVs. One study described that MERS-CoV 4b protein was a virulence factor, since deletion of 4b from the viral genome completely attenuated the virus in hDPP4-KI mice, without significant changes in the viral titers between WT and Δ4b virus in the lungs of mice. 4b protein inhibited autophagy both *in vivo*, in hDPP4-KI mice, and in the MRC5 human lung cell line (Bello-Perez et al., 2022). Additionally, deletion of 4b induced a significantly lower inflammatory response in the lungs of mice and in MRC5 cells, suggesting a link between autophagy and inflammation, with consequences in virus virulence (Bello-Perez et al., 2022). In fact, inhibition of autophagy increased the expression levels of pro-inflammatory cytokines in MERS-CoV infected cells (Bello-Perez et al., 2022). Autophagy has important effects on the induction and modulation of inflammatory processes. The cross-talk between inflammation and autophagy is an emerging field with implications for understanding viral pathogenesis and to identify potential antiviral targets (Netea-Maier et al., 2016). In another paper, MERS-CoV 4b and 5 proteins were shown to inhibit autophagy in Vero cells in the context of infection, using recombinant viruses in which either ORF4b or ORF5 had been deleted (Gassen et al., 2019). Inhibition of autophagy by 4b and 5 proteins was related with increased viral growth, suggesting that autophagy activation might also have an antiviral effect in MERS-CoV infection of Vero cells (Gassen et al., 2019).

## Endoplasmic reticulum stress pathway

The endoplasmic reticulum (ER) plays a critical role in the synthesis, folding and structural maturation of most cellular proteins. ER folding capacity depends on ER size and secretory potential, which are different in each cellular type. The ER stress occurs when the ER protein-folding capacity is saturated, leading to misfolded protein accumulation and activation of the unfolded protein response (UPR) (Oakes and Papa, 2015). The UPR is initiated by three ER transmembrane proteins that activate different pathways. The inositol-requiring enzyme 1α (IRE1α), the pancreatic endoplasmic reticulum kinase (PERK) and the activating transcription factor 6 (ATF6) pathway. The main function of the UPR is to restore the cellular homeostasis by increasing ER biogenesis and transcription of protein-folding chaperones. Alternatively, when the ER-stress is not resolved, the prolonged unfolded protein response induces programmed cell death or apoptosis (Frakes and Dillin, 2017).

Commonly, ER stress induced by viral infections leads to UPR (Mehrbod et al., 2019). Human CoVs encode multiple proteins that localize in the ER and have been related to the activation of this stress pathway, among them there are some accessory proteins (Fung et al., 2014). SARS-CoV ORF3a induces ER stress when overexpressed in Huh7 cells, as determined by the increase of grp78 transcription levels, a crucial ER chaperone. This work suggests that ORF3a specifically activates PERK, but not IRE1α or ATF6 branches of the unfolded-protein response, as determined by the increase of phosphorylated eIF2α levels (Minakshi et al., 2009). SARS-CoV ORF8b also triggered ER stress by forming intracellular aggregates, which upregulated the transcription factor CHOP, although the specific branch responsible for UPR activation still remains unclear (Shi et al., 2019). Among SARS-CoV-2 accessory proteins, ORF3a has also been related to ER stress. Although the mechanism is not clear, ORF3a overexpressed in HeLa cells activated the three UPR branches (Su WQ. et al., 2021). In addition, SARS-CoV-2 ORF3a has been reported to induce ER stress by activating BECN1 and RETREG1-dependent reticulophagy, an ER degradation process via autophagy. A549 cells transfected with ORF3a or infected with SARS-CoV-2 demonstrated to induce reticulophagy and the subsequent ER stress only in the presence of active BECN1 or RETREG1. These results suggest a new mechanism by which SARS-CoV-2 might induce ER stress in a reticulophagy dependent manner (Zhang X. et al., 2022). In addition, this same study includes results suggesting that autophagy could increase SARS-CoV-2 replication, as overexpression of ORF3a not only induces autophagy, but also increases the expression levels of viral genes S, N and RdRp (Hou et al., 2022).

## Concluding remarks

Coronavirus accessory genes are not strictly required for viral replication, although some of them may contribute to virus growth to some extent. Several CoV accessory proteins have structural functions and participate in virion structure, like SARS-CoV ORFs 3a, 3b, 6, 7a or 7b (Liu et al., 2014). However, they are mostly involved in virus virulence by interfering with the host antiviral responses. Despite their diversity in sequence and mechanism of action, CoV accessory proteins contribute to virus pathogenesis by modulating the innate immune response, the host immune system or other pathways involved in host resistance to viral infections, like autophagy or apoptosis.

The virulence of highly pathogenic human CoVs, SARS-CoV, MERS-CoV and SARS-CoV-2 is associated with lung immunopathology, characterized by massive inflammatory cell infiltration and elevated pro-inflammatory cytokine and chemokine responses. As described in this review, experimental infections of animal models with deletion mutants of accessory genes have confirmed the relevance of these genes in virus-induced immunopathology.

Attenuation of human CoVs due to virus evolution may involve the loss of virus virulence factors, including accessory genes, as observed with deletions within ORF8 during SARS-CoV epidemic (Oostra et al., 2007) or in ORFs 7 and 8 in the current SARS-CoV-2 pandemic (Young et al., 2020). These mutations, leading to defects in the suppression of the host immune response, might confer an evolutionary disadvantage, which could explain why these mutations disappear in the immunocompetent population. Alternatively, mutations in CoV accessory genes that modulate the innate immune response and may enhance viral pathogenicity have also been identified (Hassan et al., 2022). The emergence of novel human CoVs that cross the species barrier from animal hosts may involve, not only significant changes in viral proteins required for cell entry (Forni et al., 2021; Montoya et al., 2021), but also in accessory proteins that modulate the innate immune response of the new host. Similarly, a 29-nt deletion in SARS-CoV ORF8, which split ORF8 into ORF8a and ORF8b, occurred soon after its zoonotic transmission from civets to humans and was considered a virus adaptation to humans (Oostra et al., 2007). MERS-CoV accessory genes also seem to be relevant to the interaction of the virus with specific hosts. MERS-CoV ORF5 is conserved in field isolates from camels and humans, while it is highly unstable in cell cultures, with the appearance of mutations and deletions leading to a truncated protein 5 (Gutierrez-Alvarez et al., 2021). Moreover, the identification of a variety of deletions in MERS-CoV ORF3 and ORF4b in samples from camels suggest that the virus is not fully adapted to this host yet (Chu et al., 2018; Peiris and Perlman, 2022). In addition, an *in-vitro* study of MERS-CoV persistent infection in bat cells showed that ORF5 acquired a 341 nt-deletion after 15 passages (Banerjee et al., 2020).

To understand the relevance of accessory genes in pathogenesis in the context of infection, *in vivo* assays with syngenic viruses just differing in the gene of interest are essential. The availability of animal models of infection and reverse genetics systems to engineer the CoV genome is crucial for these studies.

Overexpression of individual accessory genes, in the absence of infection, provides a complementary approach to identify or add further insight into the molecular mechanism of action of accessory proteins in virus-host interactions. However, it is important to consider that the ectopic overexpression of a single protein has a different biological impact on the host cell than infection with a virus. The overexpressed protein may be localized to different subcellular compartments and establish distinct interactions in the absence of the other viral components, with different functional consequences in virus-host interactions. To date, only a very limited number of studies about CoV accessory genes have been performed in the context of *in vivo* infection (Castaño-Rodriguez et al., 2021; Gutierrez-Alvarez et al., 2021; Silvas et al., 2021; Bello-Perez et al., 2022). Further analyses are required to determine the relevance of accessory genes in virus pathogenesis and to define their mechanism of action. This information will be helpful to identify new therapeutic interventions that ameliorate the severity of coronavirus diseases.

## Author contributions

Conceptualization, MB-P and IS. Funding acquisition, LE and IS. Writing—original draft preparation, JH-T, RR-P, MB-P and IS. Writing—review and editing, JH-T, RR-P, LE, MB-P and IS. All authors contributed to the article and approved the submitted version.
